# Evaluation of port competitiveness along China’s “Belt and Road” based on the entropy-TOPSIS method

**DOI:** 10.1038/s41598-023-42755-1

**Published:** 2023-09-21

**Authors:** Jie Wang, Lilian Mo, Zhu Ma

**Affiliations:** 1 School of Economics & Management, South China Normal University, Guangzhou, 510006 China; 2 School of Port and Shipping Management, Guangzhou Maritime University, Guangzhou, 510725 China

**Keywords:** Environmental social sciences, Ocean sciences

## Abstract

Based on the literature on the factors influencing port competitiveness, this paper evaluates the competitiveness of Chinese ports along the Belt and Road based on Porter’s diamond model. The evaluation system includes four primary indicators, including port infrastructure, port operation scale, port hinterland economic level and port development potential, and 13 secondary indicators. Then, based on the entropy-TOPSIS method, an evaluation model of port competitiveness is constructed. Finally, 15 seaports along China’s “Belt and Road” are used to evaluate competitiveness, and a comprehensive ranking of port competitiveness is obtained. The advantages and shortcomings of each port are pointed out according to the evaluation results to provide a reference for the long-term development of the competitiveness of ports along the “Belt and Road” in China.

## Introduction

**Figure 1:**
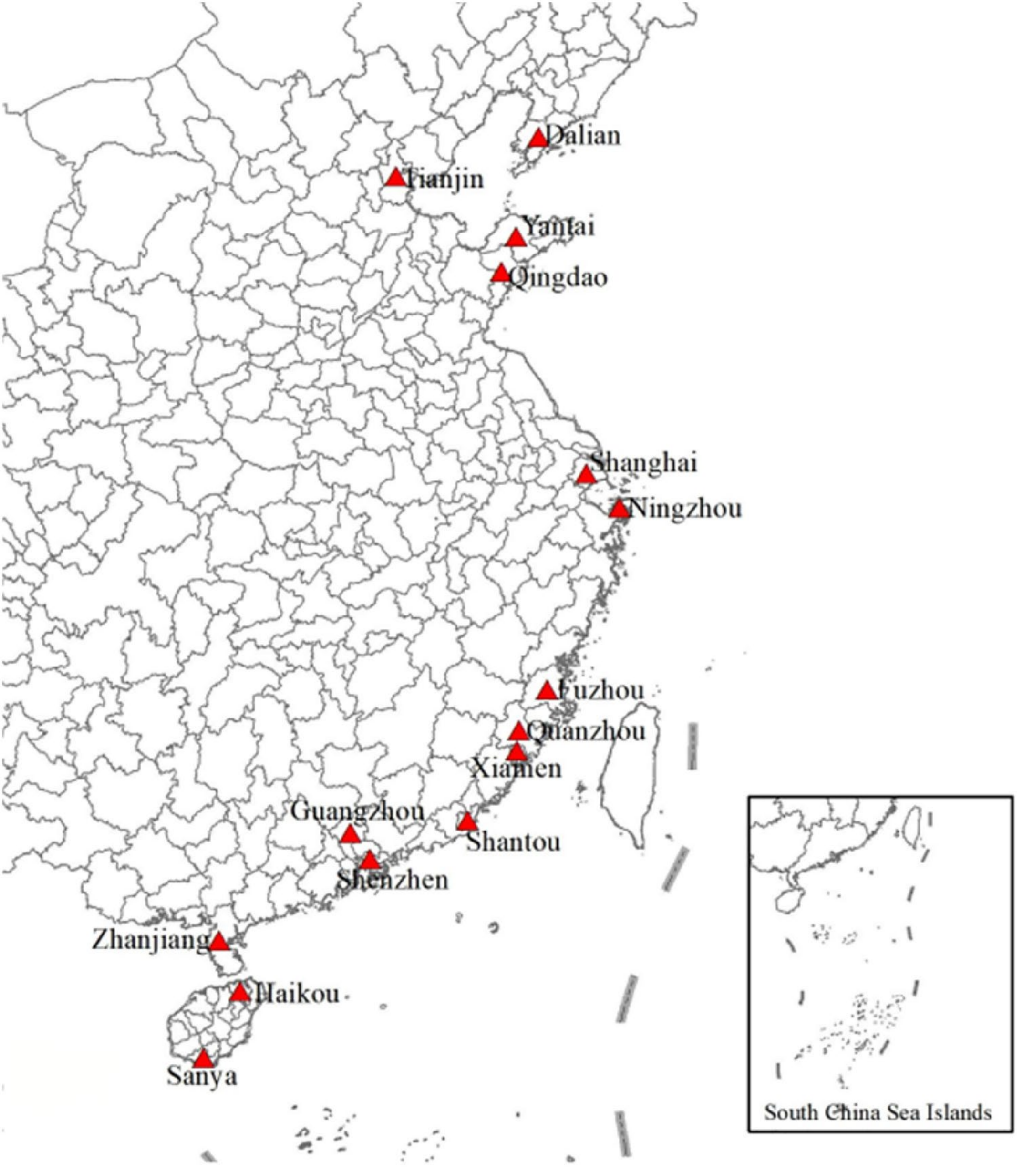
Distribution of 15 ports along China’s “Belt and Road”.

The “Belt and Road Initiative” was proposed by Chinese President Xi Jinping in 2013, and 2023 coincides with the tenth year of the “Belt and Road Initiative”. In 2015, the Chinese government further released the “Promoting the Construction of the Silk Road Economic Belt and the 21st Century Vision and Actions for the Maritime Silk Road”, which clearly pointed out the general idea of the “Belt and Road” and particularly emphasized “strengthening Shanghai, Tianjin, Ningbo-Zhoushan, Guangzhou, Shenzhen, Zhanjiang, Shantou, Qingdao, Yantai, Dalian, Fuzhou, Xiamen, Quanzhou, Haikou, Sanya and other coastal cities to build ports”. The specific locations of the relevant ports are shown in Fig. 1. Among the 15 ports along the “Belt and Road”, most of them rank among the world’s top ports in terms of throughput, and some of them have even climbed to the leading position in the top 10, such as Ningzhou Port, Shanghai Port, Tianjin Port, Guangzhou Port and Qingdao Port. The Chinese government emphasizes that in the process of promoting the construction of the “Belt and Road”, the construction of ports will be insisted upon as the pioneer, and the relevant ports will be made into the leading geese so that they can become the hubs connecting the local economic circle and the overseas economic circle.

In Chinese history, the rise of the “Maritime Silk Road” was directly related to the development of coastal ports. Currently, as the Chinese government vigorously promotes the “Belt and Road”, ports along the route play an even more important and critical role. The 15 ports along the “Belt and Road” explored in this paper occupy an extremely important strategic position among the large number of ports in China. As shown in Table 1, China is rich in port resources, with 872 ports in total, including 81 seaports. There were 22,142 berths in the country, of which 5461 were seaports and 16,681 were inland ports. The ports along the Belt and Road discussed in this paper are the best of the ports. As shown in Table 1, there are 2592 berths of 10,000 tons and above in China, among which 2138 berths of 10,000 tons and above are in seaports, accounting for 82.48%, while 1224 berths of 10,000 tons and above are in seaports, accounting for 47.22% of the total number of 10,000 tons berths in China. The number of berths above 100,000 tons in Chinese ports is 440, accounting for only 16.98% of the total number of berths, of which 97.27% are in seaports, all of which are located in ports along the “Belt and Road”. With such a distribution of ports, the evaluation and analysis of the competitiveness of Chinese ports along the “Belt and Road” will actually provide an understanding of the competitiveness of China’s core key port resources, which will also help to provide a reference for the good use of China’s port resources.

**Table 1:** Distribution of berths of 10,000 tons and above in Chinese ports in 2020.

Berth tonnage	National ports	Percentage	Seaports	Percentage	Other ports	Percentage
Total	2592	100.00	2138	82.48	454	17.52
10,000–30,000 tons	865	33.37	672	77.69	193	22.31
30,000–50,000 tons	437	16.86	313	71.62	124	28.37
50,000–100,000 tons	850	32.79	725	85.29	125	14.71
100,000 tons and above	440	16.98	428	97.27	12	2.73

*Data sources* China Ports Yearbook of 2021.

## Literature review

### Study on the factors influencing port competitiveness

Research on port competitiveness first started in the 1960s, gained widespread attention after 1980, and currently remains a topical issue in academic and industry research. The most widely used definition of port competitiveness is that of Heaver^1^. Subsequent studies on the factors influencing port competitiveness can also be based on the above definition, with more diverse subdimensions and perspectives according to the two main aspects of port facilities hardware and port services software. For example, De Martino and Morvillo^2^ divide port competitiveness criteria into hard criteria and soft criteria. Chang et al.^3^ report that the main determinants of port competitiveness are physical and operational capacity. Kim^4^ used the TOPSIS method to compare port competitiveness between a sample of Korean and Chinese ports.

Other scholars have expanded the factors influencing port competitiveness based on port facilities hardware and port services software. For example, Wan et al.^5^, Yeo et al.^6^, Dyck and Ismael^7^ listed related factors affecting port competitiveness. Bichou et al.^8^ concluded from his survey that despite the traditional conflicting relationships between international shipping and logistics channel members, it is necessary to open up information sharing channels between different channel members. Navickas et al.^9^ believed that the effectiveness of the port logistics system can be reflected by the appropriate allocation of human and other relevant resources, the competence of participants, the diversity of decision alternatives, and the decision-making process. Lakhmas^10^ states that the international competitiveness of ports depends on the efficiency and effectiveness factors of their countries.

There are also many scholars who have taken different stakeholder perspectives and found that there are relatively large differences in the factors influencing port competitiveness as defined by different stakeholders. Wiegmans et al.^11^, from the perspective of container carriers, argue that the most central element affecting the competitiveness of ports is the availability of the hinterland. Ha^12^ classifies seven factors of port selection from the carrier’s perspective. Kaliszewski^13^ analyses the factors influencing port competitiveness from the perspective of global liner shipping lines. Sedat and other scholars^14^ compare the factors influencing port competitiveness from both container terminal operators and liner companies.

### Study related to the evaluation method of port competitiveness

Regarding the relevant factors affecting port competitiveness, scholars have used various research methods to ensure the accuracy of research. Some scholars^15–17^ have used a Likert scale combined with expert ratings to determine the most important factors affecting port competitiveness. Most scholars, on the other hand, used an extensive literature review to identify the possible influencing factors of port competitiveness, such as Sedat et al.^14^, who summarized the factors defining port competitiveness through a comprehensive review of relevant literature in anonymously reviewed journals spanning more than 20 years. In addition, based on the extensive literature reading to identify possible influencing factors, principal component analysis^18,19^, cluster analysis^20^, and regression analysis^21–23^ are also used to clarify the main factors affecting the competitiveness of ports.

Many scholars have used DEA to analyze the competitiveness of ports. For example, Parola^24^ evaluated the operational efficiency and competitiveness of ports by measuring the inputs and outputs of ports in the DEA model, thus indicating aspects for the improvement of port operational efficiency. Through the analysis of five large international ports in Asia, Yang et al.^25^ concluded that operational efficiency is related to the size of the port, which usually produces high-quality infrastructure, storage, and cargo handling facilities. Additionally, by evaluating the DEA approach, Hales et al.^26^ found that reputation does not affect competitiveness. Scholar^27^ used a network-DEA centralized efficiency model to simultaneously optimize two phases of efficiency in two Brazilian ports. Scholars^28^ used a data envelopment analysis prediction model to effectively measure and predict the future performance of the port industry for 53 Vietnamese ports. The technique was also used to obtain performance indicators and establish a benchmark learning tool for ports, which provides a reference for improving the efficiency of ports.

In addition to the above studies, researchers^29^ reviewed the development of green ports in Thailand and proposed the evaluation criteria and environmental performance indicators of green ports using the example of Lien Chabang Port. Ziaul et al.^30^ proposed an approach to predict the competitiveness of transshipment ports based on market share, using a multicriteria decision making (MCDM) approach to explore the potential causes of rising or falling port competitiveness.

### Study related to the evaluation of ports along the “Belt and Road”

With the proposal of the “Belt and Road” initiative, research on ports along the “Belt and Road” has also received the attention of scholars. Among them, Wang Chuanxu et al.^31^ introduced the concept of sustainable development capability and applied principal component analysis and the analytic hierarchy process to evaluate the indicators of 15 ports along the Belt and Road in China in four dimensions, including the capacity of port operations, economic conditions, environmental factors, and human intellect and technology. Xu et al.^32^ constructed a network evolution model based on complex network theory and node attraction by selecting “Belt and Road” ports along the line in 2017 and verified the effectiveness of the model by comparing the network eigenvalues of the evolved network and the real network. Mou Naixia et al.^33^ evaluated the location advantages of the ports along the Maritime Silk Road from a wider perspective. They selected ports in 63 countries along the Maritime Silk Road.

### Summary of the literature

From the literature on the analysis of factors influencing port competitiveness, much of the literature discusses some of the important influencing factors, including port infrastructure, operational factors, the economic situation of the port’s hinterland, and the future development potential of the port. However, there is little literature that comprehensively analyses the above four factors, and there is no literature that constructs an analytical framework for these influencing factors based on Porter’s Diamond Model, which is one of the innovations of this paper.

In terms of research methods, some studies, such as the literature^15–17^, sorted out relevant port competitiveness influencing factors through a literature review and then adopted an expert scoring method to assess these factors, in which the expert assessment is subjective to some extent. Many studies^24–28^ use data envelopment analysis to measure port competitiveness enhancement. In our study, entropy-TOPSIS is a comprehensive evaluation method and is a common multiobjective decision analysis method that is applicable to the comparative study of multiple solutions and objects, from which the best solution or the most competitive object can be identified. For the above reasons, the entropy-TOPSIS method is an objective and appropriate choice for this paper.

## Methodology

### Indicator system construction

The diamond model proposed by management scientist Michael Porter is often used as a theoretical basis for studying industrial competitiveness. According to Porter, the ability of a country or region to have international competitive advantage in a certain industry depends on four key factors, which interact with each other to form a two-way reinforcing diamond system.

Taking the factors of production in Porter’s diamond model as an example, the most important factor of production for ports is infrastructure, and the existing research literature on the factors influencing port competitiveness, such as studies^1,2,4,5,7,8,10,12–14^, all elaborate on the importance of port infrastructure for enhancing port competitiveness. Given the availability of data, the data we can obtain for the infrastructure of 15 ports along the Belt and Road (P1) include the total number of berths (S11), the number of 10,000-ton berths (S12), and the total length of terminals (S13). Some important port facilities, such as IT systems, are recognized in the literature and by the authors of this paper as important to port competitiveness, but because standardized public data on IT systems in Chinese ports are not available, the port production factors of this paper’s evaluation index system do not include IT systems, which is a shortcoming of this paper caused by the data.

Based on the above similar ideas, this paper starts from Porter’s diamond model, locates the relevant indicators through the literature, while taking into account the data availability, and thus constructs a port competitiveness evaluation index system, as shown in Table 2.

**Table 2:** Port competitiveness indicators based on the Diamond Model.

Target level	Code level	Primary indicators	Secondary indicators	References
Port competitiveness	Port production factors	Port infrastructure (P1)	Total number of berths (S11)	^1,2,4,5,7,8,10,12–14^
			Number of 10,000-ton berths (S12)	
			Total quay length (S13)	
	Port demand conditions	Port operation scale (P2)	Cargo throughput (S21)	^2,3,5–7,12–14^
			Container throughput (S22)	
			Foreign trade cargo throughput (S23)	
	Port related and supporting industries	Economic level of port hinterland (P3)	Total city GDP (S31)	^6,11,12,14^
			City tertiary industry output value (S32)	
			City’s foreign trade volume (S33)	
	Port strategy, structure and competition	Port development potential (P4)	Cargo throughput growth rate (S41)	^3,4^
			Growth rate of container throughput (S42)	
			City GDP growth rate (S43)	
			Growth rate of city’s foreign trade volume (S44)	

In the subsequent charts, to save space, P1 to P4 will be used to represent the four primary indicators, and S11, S12,…, S44 will be used to represent the 13 secondary indicators, as shown in parentheses in Table.

### Port competitiveness of entropy-TOPSIS

The entropy weighting is an objective assignment model. The TOPSIS method is the ranking method that approximates the ideal value. Based on the Diamond Model port competitiveness evaluation index system, this paper uses entropy-TOPSIS analysis to model and analyze all the indexes. The following are the specific steps.

*Step 1* Determine the original data matrix. $x_{ij}$ is the observed value of the jth evaluation indicator of the ith port, then the evaluation matrix of each port and the raw data corresponding to each evaluation indicator is $X = \left[ {x_{ij} } \right]_{n \times m}$. The specific evaluation matrix is as follows.

1 $$\begin{aligned} X & = \left[ {x_{{ij}} } \right]_{{n \times m}} = \left[ {\begin{array}{*{20}c} {x_{{11}} } & {x_{{12}} } & {x_{{13}} } & \cdots & {x_{{1m}} } \\ {x_{{21}} } & {x_{{22}} } & {x_{{23}} } & \cdots & {x_{{2m}} } \\ \vdots & \vdots & {} & \ddots & {} \\ {x_{{n1}} } & {x_{{n2}} } & {x_{{n3}} } & \ldots & {x_{{nm}} } \\ \end{array} } \right], \\ i & = 1,2,3, \ldots ,n;j = 1,2,3, \ldots ,m. \\ \end{aligned}$$

*Step 2* Dimensionless processing of the corresponding raw port data. Since different indicators have different units, the evaluation results can be biased. Therefore, it is necessary to remove the dimensionality of all indicator data to ensure the validity of the evaluation. The extreme difference method is applied to obtain the standardization matrix Y.

The formula for normalizing the extreme difference of positive indicators is as follows:

2 $$y_{ij} = \frac{{x_{ij} - x_{j\min } }}{{x_{j\max } - x_{j\min } }}.$$

The formula for normalizing the extreme difference of negative indicators is as follows:

3 $$y_{ij} = 1 - \frac{{x_{ij} - x_{j\min } }}{{x_{j\max } - x_{j\min } }}.$$

The standardization matrix is:

4 $$Y = \left[ {y_{ij} } \right]_{m*n} { = }\left[ {\begin{array}{*{20}c} {y_{11} } & {y_{12} } & {y_{13} } & \cdots & {y_{1m} } \\ {y_{21} } & {y_{22} } & {y_{23} } & \cdots & {y_{2m} } \\ \vdots & \vdots & {} & \ddots & {} \\ {y_{n1} } & {y_{n2} } & {y_{n3} } & \ldots & {y_{nm} } \\ \end{array} } \right].$$

*Step 3* Determine the weight of each port indicator. Calculate the weight $p_{ij}$ of the ith port under the jth indicator in the port competitiveness model. The formula for calculating the weight $p_{ij}$ is:

5 $$p_{ij} = \frac{{y_{ij} }}{{\sum\nolimits_{i = 1}^{n} {y_{ij} } }}.$$

*Step 4* Calculate the entropy value of each evaluation index of port competitiveness. The formula is:

6 $$e_{j} = - \frac{1}{\ln n}\sum\limits_{i = 1}^{n} {p_{ij} \ln } p_{ij} .$$

*Step 5* Calculate the coefficient of variability of the first indicator. The formula is:

7 $$g_{j} = 1 - e_{j} .$$

*Step 6* Calculate the weight of the jth evaluation index. The formula is:

8 $$w_{j} = \frac{{g_{j} }}{{\sum\nolimits_{j = 1}^{n} {g_{j} } }}.$$

*Step 7* Determine the positive and negative ideal solution vectors. The formula is:

9 $$Y^{ + } = \left( {\max y_{i1} ,\max y_{i2} ,\max y_{i3} ,...,\max y_{im} } \right),$$

10 $$Y^{ - } = \left( {\min y_{i1} ,\min y_{i2} ,\min y_{i3} ,...,\min y_{im} } \right).$$

*Step 8* Calculate the weighted distance. The formula is:

11 $$D_{i}^{ + } = \sqrt {\sum\limits_{j = 1}^{m} {w_{j} \left( {\max y_{ij} - y_{ij} } \right)^{2} } } ,$$

12 $$D_{i}^{ - } = \sqrt {\sum\limits_{j = 1}^{m} {w_{j} \left( {\min y_{ij} - y_{ij} } \right)^{2} } } .$$

*Step 9* Calculate the competitiveness score of each port. Let $B_{i}$ be the evaluation value of competitiveness of the ith port. The specific calculation formula is:

13 $$B_{i} { = }\frac{{D_{i}^{ - } }}{{D_{i}^{ + } + D_{i}^{ - } }}.$$

In this formula, the larger $B_{i}$ is, the better the state of the comparison object. If all the indicators of the comparison object are in the best state, $B_{i} = 1$; if all the indicators of the comparison object are in the worst state, $B_{i} = 0$. After that, all the evaluation objects are ranked according to the value of relative closeness $B_{i}$. The closer $B_{i}$ is to 1, the better the performance of the corresponding evaluation object; otherwise, the worse the performance. The result of ranking all the ports is thus obtained, and the competitiveness of all the ports is evaluated according to this result.

## Results

### Data

Based on the strategic importance of the 15 ports along the “Belt and Road” in China, which are described in the introduction section, this paper evaluates and analyses the competitiveness of these 15 ports. According to the evaluation model, which was constructed in the previous section, the original data of 13 secondary indexes of ports along the “Belt and Road” were collected in Table 3.

**Table 3:** Data of indicators for each port.

Port	P1			P2			P3			P4			
	S11	S12	S13	S21	S22	S23	S31	S32	S33	S41	S42	S43	S44
Shanghai	1024	185	105,814	71,670	4350	38,917	38,701	28,308	87,463	− 0.01	0.01	0.02	0.04
Tianjin	192	117	41,776	50,290	1835	28,500	14,084	9069	7341	0.02	0.06	0.02	0.00
Ningzhou	699	196	100,955	117,240	2872	53,679	13,921	7145	18,248	0.05	0.04	0.04	− 0.01
Guangzhou	621	80	50,011	63,643	2351	14,361	25,019	18,141	9530	0.02	0.01	0.03	− 0.05
Shenzhen	168	75	34,036	26,506	2655	18,897	27,670	17,190	30,503	0.03	0.03	0.03	0.02
Zhanjiang	159	42	22,878	23,391	123	9644	3100	1426	442	0.08	0.10	0.02	0.07
Shantou	34	11	5013	3351	159	1056	2731	1304	682	0.06	0.18	0.02	0.14
Qingdao	119	89	30,625	60,459	2201	44,458	12,401	7614	6407	0.05	0.05	0.04	0.08
Yantai	223	107	39,294	39,935	330	14,414	7816	3192	3220	0.03	0.06	0.04	0.11
Dalian	256	111	47,095	33,401	511	16,350	7030	3756	3854	− 0.09	− 0.42	0.01	− 0.12
Fuzhou	158	67	26,233	19,944	338	5477	10,020	5619	2505	0.17	− 0.01	0.05	− 0.01
Xiamen	175	78	31,476	20,750	1141	10,216	6384	3835	6916	− 0.03	0.03	0.06	0.08
Quanzhou	94	52	22,465	11,805	226	3767	10,159	4124	1971	− 0.07	− 0.12	0.03	− 0.07
Haikou	70	34	9867	11,781	197	365	1792	1442	368	− 0.05	0.00	0.05	0.11
Sanya	13	0	2768	221	0.78	0.7	695	503	172	0.12	0.01	0.03	0.77

The relevant data are obtained from the National Bureau of Statistics of China, the official websites of the General Administration of Customs of China and its direct departments, the official websites of the governments of various provinces and cities and their transport bureaus, statistical bureaus and the China Ports Yearbook (2021 edition).

### Evaluation process

#### Data standardization

According to Eqs. (1) to (4), all the raw data collected were dimensionless and resulted in the normalization matrix shown in Table 4.

**Table 4:** Standardization matrix.

Port	P1			P2			P3			P4			
	S11	S12	S13	S21	S22	S23	S31	S32	S33	S41	S42	S43	S44
Shanghai	1.00	0.94	1.00	0.60	1.00	0.72	1.00	1.00	1.00	0.32	0.71	0.17	0.62
Tianjin	0.16	0.57	0.36	0.41	0.41	0.53	0.33	0.29	0.08	0.43	0.80	0.13	0.46
Ningzhou	0.67	1.00	0.95	1.00	0.65	1.00	0.33	0.22	0.21	0.52	0.77	0.69	0.42
Guangzhou	0.59	0.37	0.45	0.53	0.53	0.26	0.63	0.62	0.11	0.40	0.72	0.38	0.27
Shenzhen	0.14	0.35	0.29	0.20	0.60	0.35	0.70	0.59	0.35	0.45	0.75	0.46	0.56
Zhanjiang	0.13	0.17	0.18	0.18	0.00	0.17	0.04	0.00	0.00	0.67	0.86	0.21	0.73
Shantou	0.00	0.00	0.00	0.00	0.01	0.01	0.03	0.00	0.00	0.58	1.00	0.23	1.00
Qingdao	0.09	0.42	0.25	0.50	0.49	0.83	0.29	0.23	0.07	0.52	0.78	0.58	0.79
Yantai	0.19	0.52	0.34	0.32	0.05	0.26	0.16	0.07	0.03	0.47	0.81	0.56	0.89
Dalian	0.22	0.54	0.42	0.26	0.09	0.30	0.14	0.09	0.04	0.00	0.00	0.00	0.00
Fuzhou	0.13	0.30	0.21	0.15	0.05	0.10	0.22	0.16	0.02	1.00	0.68	0.88	0.42
Xiamen	0.14	0.36	0.26	0.15	0.24	0.18	0.12	0.09	0.08	0.23	0.74	1.00	0.77
Quanzhou	0.06	0.22	0.17	0.07	0.02	0.06	0.23	0.10	0.02	0.06	0.49	0.42	0.20
Haikou	0.04	0.12	0.05	0.07	0.02	0.00	0.00	0.01	0.00	0.14	0.70	0.92	0.91
Sanya	0.00	0.00	0.00	0.00	0.00	0.00	0.00	0.00	0.00	0.79	0.72	0.46	1.00

#### Calculation of entropy value, variability index and weights

The entropy value of each evaluation index is obtained by substituting the standardized data into Eqs. (5) and (6). The calculated entropy value is substituted into Eqs. (7) and (8) to obtain the weights of each evaluation index. The entropy values, variability indexes and weights of each index are shown in Table 5.

**Table 5:** Entropy value, variability index and weight of each indicator.

Primary index	Secondary index	Entropy value	Variability index	Weights
P1	S11	0.81	0.19	0.10
	S12	0.91	0.09	0.05
	S13	0.89	0.11	0.06
P2	S21	0.88	0.12	0.07
	S22	0.79	0.21	0.11
	S23	0.85	0.15	0.08
P3	S31	0.85	0.15	0.08
	S32	0.78	0.22	0.12
	S33	0.63	0.37	0.20
P4	S41	0.92	0.08	0.04
	S42	0.97	0.03	0.02
	S43	0.92	0.08	0.04
	S44	0.94	0.06	0.03

Repeating the above process, we can calculate the specific weights of the indicators at each level of the port competitiveness evaluation model, as shown in Table 6. The comprehensive weights of the second-level indicators refer to the indicator weights, the weights of the first-level indicators are obtained by summing the comprehensive weights of the corresponding secondary index, and the intragroup weights of the second-level indicators refer to the weights occupied by each secondary indicator in the corresponding first-level indicator group.

**Table 6:** The specific weights of each indicator.

Primary index	Weights	Secondary index	Intragroup weights	Combined weights
P1	0.2087	S11	0.4864	0.1015
		S12	0.2189	0.0457
		S13	0.2946	0.0615
P2	0.2608	S21	0.2525	0.0658
		S22	0.4381	0.1143
		S23	0.3094	0.0807
P3	0.3966	S31	0.2077	0.0824
		S32	0.2941	0.1166
		S33	0.4982	0.1976
P4	0.1339	S41	0.3147	0.0421
		S42	0.1281	0.0172
		S43	0.3183	0.0426
		S44	0.2389	0.0320

As shown in Table 6, the indicator of P3 has the highest weight of 0.3966, which means that the economic level of the related city is important in the overall competitiveness of the port. The second is the scale of port operation (P2) and port infrastructure (P1); the weights of these two primary indicators are 0.2608 and 0.2087, respectively. The weakest primary indicator is port development potential (P4), which accounts for 0.1339.

From the standpoint of the intragroup weights of the secondary indicators in Table 6, the most important indicator in port infrastructure (P1) is the total number of berths (S11), which accounts for approximately 0.4864, followed by the total quay length (S13) and the 10,000-ton berth quantity (S12). In terms of port operation scale (P2), the indicator with the highest proportion is container throughput (S22), with a weight of approximately 0.4381, followed by foreign trade cargo throughput (S23) and cargo throughput (S21). For the economic level of related cities (P3), the most important secondary index is the total foreign trade value (S33), with a weight of approximately 0.4982, followed by the city’s third industry outputs (S32) and the city’s GDP (S31). In terms of port development potential (P4), the rates of cargo (S41) and city GDP (S43) have almost equal shares, which are 0.3147 and 0.3183, respectively; the next most important indicators are the city’s foreign trade volume (S44) and the container throughput rate (S42), respectively.

#### Calculation of weighted distance, relative closeness and competitiveness ranking

The calculated distances, relative closeness and ranking of competitiveness of each port with respect to the optimal and the worst values are shown in Table 7.

**Table 7:** Weighted distance, relative proximity and competitiveness ranking of each port.

Port	Distance to the best solution	Distance to the worst solution	Relative closeness	Ranking
Shanghai	0.34	0.87	0.72	1
Tianjin	0.72	0.35	0.33	6
Ningzhou	0.55	0.64	0.54	2
Guangzhou	0.64	0.45	0.42	3
Shenzhen	0.62	0.44	0.41	4
Zhanjiang	0.88	0.22	0.20	13
Shantou	0.93	0.20	0.17	14
Qingdao	0.70	0.41	0.37	5
Yantai	0.79	0.29	0.26	10
Dalian	0.85	0.22	0.21	11
Fuzhou	0.83	0.32	0.28	7
Xiamen	0.80	0.30	0.27	8
Quanzhou	0.89	0.16	0.15	15
Haikou	0.91	0.23	0.20	12
Sanya	0.92	0.34	0.27	9

The competitiveness of each port was calculated. Next, this paper will analyze the competitiveness of Chinese ports along the Belt and Road according to the above calculation results.

### Result analyses

#### Comprehensive evaluation of port competitiveness

The calculation results of the entropy-TOPSIS method are collated to obtain the ranking of each aspect of the first-tier indicators, as shown in Table 8.

**Table 8:** The first-level indicators and competitiveness ranking of each port.

Port	Port infrastructure (P1)	Port operation scale (P2)	Economic level of the hinterland (P3)	Port development potential (P4)	Port competitiveness
Shanghai	1	2	1	11	1
Tianjin	5	4	5	12	6
Ningzhou	2	1	4	10	2
Guangzhou	3	5	3	13	3
Shenzhen	7	8	2	9	4
Zhanjiang	11	9	13	4	13
Shantou	14	14	12	2	14
Qingdao	9	3	6	5	5
Yantai	6	6	11	3	10
Dalian	4	7	9	15	11
Fuzhou	10	11	8	6	7
Xiamen	8	10	7	8	8
Quanzhou	12	12	10	14	15
Haikou	13	13	14	7	12
Sanya	15	15	15	1	9

As seen from Table 8, under the port competitiveness system constructed in this paper, the top three ports in the comprehensive ranking are Shanghai, Ningzhou, Guangzhou, which represent the three most competitive ports of “B&R” in China. Quanzhou, Shantou and Zhanjiang Port are in the bottom three in the ranking of port competitiveness and still need to improve port competitiveness in all aspects.

Looking at different indicators, in terms of port infrastructure (P1), the three most competitive ports of “B&R” are Shanghai, Ningzhou and Guangzhou Ports, while Sanya, Shantou and Haikou Ports rank the lowest in terms of port infrastructure and still have great room for construction. In terms of port operation scale (P2), the top three ports along the “Belt and Road” are Ningzhou Port, Shanghai Port, and Qingdao Port, while the bottom three are Sanya Port, Shantou Port and Haikou Port. For the economic level of the port hinterland (P3), the top three ports are Shanghai, Shenzhen and Guangzhou, while the three ports with the worst economic level of the hinterland are Sanya, Haikou and Zhanjiang. Regarding port development potential (P4), the top three ports along the “Belt and Road” are Sanya Port, Shantou Port, and Yantai Port, which show strong growth potential. Dalian, Quanzhou, and Guangzhou have the most backward development potential and limited space for future development.

#### Evaluation and analysis of port infrastructure

The raw data of the secondary indicators under the indicator of port infrastructure and the corresponding ranking of the secondary indicators are organized as shown in Table 9.

**Table 9:** Port infrastructure data and the corresponding secondary indicator rankings.

Port	Rank of P1	Data of S11	Rank of S11	Data of S12	Rank of S12	Data of S13	Rank of S13
Shanghai	1	1024	1	185	2	105,814	1
Tianjin	5	192	6	117	3	41,776	5
Ningzhou	2	699	2	196	1	100,955	2
Guangzhou	3	621	3	80	7	50,011	3
Shenzhen	7	168	8	75	9	34,036	7
Zhanjiang	11	159	9	42	12	22,878	11
Shantou	14	34	14	11	14	5013	14
Qingdao	9	119	11	89	6	30,625	9
Yantai	6	223	5	107	5	39,294	6
Dalian	4	256	4	111	4	47,095	4
Fuzhou	10	158	10	67	10	26,233	10
Xiamen	8	175	7	78	8	31,476	8
Quanzhou	12	94	12	52	11	22,465	12
Haikou	13	70	13	34	13	9867	13
Sanya	15	13	15	0	15	2768	15

In terms of port infrastructure (P1), among the ports along the “Belt and Road”, the three most competitive ports are Shanghai Port, Ningzhou Port and Guangzhou Port, mainly due to their favorable advantages in total berths, 10,000-ton berths and total quay length. As shown in Table 9, Shanghai and Ningzhou Guangzhou rank in the top 3 along the “Belt and Road” in terms of the total number of berths (S11) and total terminal length (S13), while Ningzhou Port ranks first with 196 and Shanghai Port ranks second with 185 in terms of S12.

Sanya Port, Shantou Port and Haikou Port have the lowest ranking in port infrastructure, which is also closely related to the shortage of these three ports in the three indicators of total berths, 10,000-ton berths and total quay length. Table 9 shows that Sanya Port, Shantou Port and Haikou Port are always ranked 15th, 14th and 13th in terms of total number of berths (S11), 10,000-ton berths (S12) and total length of quays (S13), which shows the disadvantageous position of these three ports in terms of infrastructure.

#### Evaluation and analysis of port operation scale

The raw data and ranking of the indicator of port operation scale and the subordinate secondary indicators of cargo throughput, container throughput and foreign trade cargo throughput are organized as shown in Table 10.

**Table 10:** Port operation scale and the corresponding secondary index ranking.

Port	Rank of P2	Data of S21	Rank of S21	Data of S22	Rank of S22	Data of S23	Rank of S23
Shanghai	2	71,670	2	4350	1	38,917	3
Tianjin	4	50,290	5	1835	6	28,500	4
Ningzhou	1	117,240	1	2872	2	53,679	1
Guangzhou	5	63,643	3	2351	4	14,361	8
Shenzhen	8	26,506	8	2655	3	18,897	5
Zhanjiang	9	23,391	9	123	14	9644	10
Shantou	14	3351	14	159	13	1056	13
Qingdao	3	60,459	4	2201	5	44,458	2
Yantai	6	39,935	6	330	10	14,414	7
Dalian	7	33,401	7	511	8	16,350	6
Fuzhou	11	19,944	11	338	9	5477	11
Xiamen	10	20,750	10	1141	7	10,216	9
Quanzhou	12	11,805	12	226	11	3767	12
Haikou	13	11,781	13	197	12	365	14
Sanya	15	221	15	1	15	1	15

As shown in Table 10, among the ports along the “Belt and Road”, the top three ports in terms of port operation scale (P2) are Ningzhou Port, Shanghai Port and Qingdao Port, while the bottom three are Sanya Port, Shantou Port and Haikou Port. From the performance of the three secondary indicators, the best performing ports of cargo throughput (S21) include Ningzhou, Shanghai, and Guangzhou; the best performing ports of container throughput (S22) are Shanghai, Ningzhou, Shenzhen Port. The best performing foreign trade cargo throughputs (S23) are Ningzhou, Qingdao, and Shanghai Port.Due to the economic level, GDP growth and foreign trade environment of the cities and regions where the ports are located, Sanya Port, Shantou Port and Haikou Port are ranked low in cargo throughput (S21), container throughput (S22), and foreign trade cargo throughput (S23), which affects their competitiveness in terms of port operation scale.

#### Evaluation and analysis of the economic level of port hinterland

The raw data and ranking of the indicator of the economic level and the subordinate secondary indicators are organized as shown in Table 11.

**Table 11:** Economic level of port hinterland data and the corresponding secondary index ranking.

Port	Rank of P3	Data of S31	Rank of S31	Data of S32	Rank of S32	Data of S33	Rank of S33
Shanghai	1	38,701	1	28,308	1	87,463	1
Tianjin	5	14,084	4	9069	4	7341	5
Ningzhou	4	13,921	5	7145	6	18,248	3
Guangzhou	3	25,019	3	18,141	2	9530	4
Shenzhen	2	27,670	2	17,190	3	30,503	2
Zhanjiang	13	3100	12	1426	13	442	13
Shantou	12	2731	13	1304	14	682	12
Qingdao	6	12,401	6	7614	5	6407	7
Yantai	11	7816	9	3192	11	3220	9
Dalian	9	7030	10	3756	10	3854	8
Fuzhou	8	10,020	8	5619	7	2505	10
Xiamen	7	6384	11	3835	9	6916	6
Quanzhou	10	10,159	7	4124	8	1971	11
Haikou	14	1792	14	1442	12	368	14
Sanya	15	695	15	503	15	172	15

As shown in Table 11, for the economic level of ports, the top three ports are Shanghai, Shenzhen and Guangzhou, while the bottom three are Sanya, Haikou and Zhanjiang. From the performance of the three secondary indicators, the best performing ports in total city GDP (S31) are Shanghai, Shenzhen and Guangzhou ports; the best performing ports in terms of city tertiary industry output (S32) are Shanghai, Guangzhou and Shenzhen ports. The three best performing ports in total foreign trade imports and exports (S33) are Shanghai, Shenzhen, and Ningzhou. Sanya, Haikou and Zhanjiang ports are always ranked low in terms of GDP value of the hinterland cities (S31), urban tertiary industry (S32) and total import and export value of the cities (S33), which affects their competitiveness in terms of the economic level of the port hinterland due to the geographical location, economic development level and foreign trade environment of the cities and regions where the ports are located.

#### Evaluation and analysis of port development potential indicators

The raw data and ranking of development potential and the four secondary indicators under it, for example, the growth rates of cargo throughput, container throughput, city GDP and city foreign trade imports and exports, are organized as shown in Table 12.

**Table 12:** Port development potential scale and the corresponding secondary index ranking.

Port	Rank of P4	Data of S41	Rank of S41	Data of S42	Rank of S42	Data of S43	Rank of S43	Data of S44	Rank of S44
Shanghai	11	− 0.005	11	0.005	11	0.017	13	0.038	8
Tianjin	12	0.022	9	0.061	4	0.015	14	− 0.001	10
Ningzhou	10	0.047	5	0.043	6	0.042	4	− 0.011	12
Guangzhou	13	0.015	10	0.012	9	0.027	10	− 0.048	13
Shenzhen	9	0.028	8	0.030	7	0.031	7	0.024	9
Zhanjiang	4	0.084	3	0.099	2	0.019	12	0.067	7
Shantou	2	0.062	4	0.180	1	0.020	11	0.135	2
Qingdao	5	0.047	5	0.047	5	0.037	5	0.082	5
Yantai	3	0.034	7	0.064	3	0.036	6	0.108	4
Dalian	15	− 0.088	15	− 0.417	15	0.009	15	− 0.117	15
Fuzhou	6	0.171	1	− 0.008	13	0.051	3	− 0.010	11
Xiamen	8	− 0.028	12	0.025	8	0.057	1	0.078	6
Quanzhou	14	− 0.072	14	− 0.124	14	0.029	8	− 0.067	14
Haikou	7	− 0.053	13	− 0.001	12	0.053	2	0.112	3
Sanya	1	0.115	2	0.010	10	0.031	7	0.766	1

As shown in Table 12, among the ports along the Belt and Road, the top three ports in terms of development potential (P4) are Sanya, Shantou, and Yantai, while Dalian Port, Quanzhou Port and Guangzhou Port rank at the bottom in terms of development potential (P4).

From the performance of the four secondary indicators, the best performing ports in terms of cargo throughput rate of increase (S41) are Fuzhou Port, Sanya Port and Zhanjiang Port; the three best performing ports in terms of container throughput rate of increase (S42) are Shantou, Zhanjiang and Yantai Port; and the three best performing ports in terms of city GDP growth (S43) are Xiamen, Haikou and Fuzhou. The best performing ports in terms of the city foreign trade import and export growth rate (S44) are Sanya, Shantou Port and Haikou Port.

In Table 12, we can find that in the four secondary indicators of cargo throughput rate of increase (S41), container throughput rate of increase (S42), city GDP rate of increase (S43) and city foreign trade import and export value growth rate (S44), Dalian, Quanzhou, Guangzhou, and even Shanghai Port are all ranked relatively low, which is very different from the larger competitive advantages shown by these ports in other indicators. We think this is mainly because the port development potential and the four subordinate indicators, including the rate of increase of cargo throughput, container throughput, city GDP, and city foreign trade import and export volume, are all growth rate indicators. Guangzhou Port, Shanghai Port, and Dalian Port, due to the previous cargo throughput container throughput, city GDP, and city foreign trade volume of the absolute value, are relatively large; therefore, it is more difficult to improve on the basis of the larger absolute value, which leads to the lower ranking of Dalian Port, Quanzhou Port, Guangzhou Port and even Shanghai Port in the indicators related to port growth potential. In contrast, Sanya, Shantou Port and Yantai Port ranked high in terms of port growth potential, possibly because, on the other hand, the base is small and easy to increase.

## Conclusions

In this paper, on the basis of the relevant literature on the factors influencing the competitiveness of ports, an evaluation system of port competitiveness is constructed based on Porter’s diamond model.

This study has important policy implications for the development of ports along China’s “Belt and Road”. On the one hand, it points the way to improve the competitiveness of Chinese ports along the “Belt and Road”. Taking the top three ports in terms of competitiveness, Shanghai Port, Ningzhou Port and Guangzhou Port, for example, although these three ports are currently ranked high in competitiveness among the 15 “Belt and Road” ports, they still have an obvious common shortcoming, i.e., none of them has a high P4 ranking, and these three ports in the top three overall competitiveness rank only 11th, 10th and 13th in terms of development potential. This is certainly related to the fact that it is more difficult for the leading ports to continue to grow, but if these three ports want to maintain a sustainable competitive advantage, they still need to focus on port development potential in the future. At the same time, the Chinese government and relevant management authorities should also focus on ports that rank low in the competitiveness assessment, such as Quanzhou Port, Shantou Port, Zhanjiang Port and Sanya Port, which rank low in many aspects of the competitiveness assessment. Although policymakers cannot completely change the existing situation, they can make targeted policy tilts, such as increasing infrastructure investment in these ports, to improve their port competitiveness. In addition, in order to enhance the competitiveness of ports along the Belt and Road, port operators should enhance their service consciousness and establish a customer-centered concept. Most of the Chinese port operators are transformed from state monopoly and government operation, so the service consciousness and customer-centered consciousness are relatively weak, and it is urgent for the port operators to change the traditional state monopoly thinking and establish the service consciousness in order to enhance the competitiveness of the port operation from the concept of thinking; Meanwhile, in today’s fast-changing science and technology, port operators along the “Belt and Road”, who are committed to improving their competitiveness, need to follow the trend of globalization, improve the level of hardware and software, develop port products in line with the world level, and improve the speed and efficiency of port services.

Although this paper has conducted a relatively detailed evaluation and analysis of port competitiveness along China’s “Belt and Road”, there are still shortcomings. First, since the factors affecting the competitiveness of ports are changing dynamically, if we want to ensure the dynamic validity of the evaluation system of port competitiveness, we must consider the current market, the immediate demand of the port and the economic development environment to improve the formation of a dynamic evaluation system of port competitiveness. For subsequent research, it might be more enriching to accentuate the use of real-time or frequently updated datasets. Furthermore, the inclusion of machine learning or predictive analytics tools might be beneficial in capturing the dynamism inherent in such factors. Second, while the current analytical framework of this paper is relatively exhaustive, given the availability of data, there is still abundant room to take into account external geopolitics, trade-centered influences, port reputation, governance changes, and green development that may affect the Belt and Road Initiative and thus port competitiveness.

## Data Availability

The datasets are available from the corresponding author on reasonable request.
